# A Prospective Cohort Study of the Therapeutic Patterns, Challenges and Outcomes of Paediatric Femoral Fractures in a Cameroonian Tertiary Center

**DOI:** 10.2174/1874325001711010029

**Published:** 2017-02-14

**Authors:** Joel Noutakdie Tochie, Marc Leroy Guifo, Marie-Ange Ngo Yamben, Roger Moulion, Ibrahim Farikou

**Affiliations:** 1 Department of Surgery and Sub-specialties, Faculty of Medicine and Biomedical Sciences, University of Yaoundé 1, Yaoundé, Cameroon; 2 Department of Surgery, University Teaching Hospital of Yaoundé, Yaoundé, Cameroon; 3 Department of Orthopaedics and Traumatology, Yaoundé Rehabilitation Centre, Yaoundé, Cameroon; 4 Department of Radiology and Medical Imaging, University Teaching Hospital of Yaoundé, Yaoundé, Cameroon

**Keywords:** Femoral fractures, Paediatric, Treatment, Challenges, Outcomes, Cameroon

## Abstract

**Background:**

Knowledge of the therapeutic patterns, challenges and outcomes of treatment of paediatric femoral fractures (PFF) helps to better choose the ideal therapeutic modality which is still controversial. However, this data is scarce in the sub-Saharan African literature.

**Objective:**

To determine the therapeutic patterns, treatment challenges and outcomes of treatment of PFF in a tertiary care centre in Cameroon.

**Method:**

We conducted a prospective cohort study of all consenting consecutive cases of femoral fractures in patients younger than 16 years managed between 2011 and 2015 at the surgical unit of Yaoundé University Teaching Hospital, Cameroon. We analysed demographic data, injury characteristics, fracture patterns, treatment details, therapeutic challenges and outcomes of treatment at 12 months using Flynn’s criteria.

**Results:**

We enrolled 30 femoral fractures from 29 children with mean age was 4.2 ± 3.3 years. The male gender, diaphyseal locations and spiral fracture lines were predominant. Main mechanisms of injury were accidental falls, road traffic accidents and game injuries. Fracture management entailed 12 tractions followed by casting, 10 casting alone, four closed reductions followed by casting, two cannulated screw fixations, one pin fixation and one external fixation. The mean duration of consolidation was 10.3 ± 3.9 weeks. The outcome was rated excellent in 28 cases. Limited resources precluded fluoroscopy use, proper anaesthetic management, early rehabilitation and patient-parent satisfaction.

**Conclusion:**

Conservative management of PFF yields a good outcome in our setting. However, an improvement in surgical, radiology and anaesthetic infrastructure is needed for optimal PFF care.

## INTRODUCTION

Femoral fractures occur at a rate of 20 per 100,000 children in the USA, representing 1.6% of all paediatrics fractures [1], yet they inflict significant adverse physical, social, psychological, and financial impacts to both affected children and parents [2]. They are more common amongst males [1, 3]. Their incidence is bimodal: first peaking between two and four years and later during adolescence [3, 4]. The aetiologies are often age-dependent and include traffic accidents, unintentional injuries (falls and games), child abuse and pathological states [3, 4].

There is still no consensus on the best method of managing paediatric femoral fractures, especially diaphyseal fractures. Evidence from a recent systematic review of randomized controlled trials did not ascertain the efficacy of conservative management over surgical management of femoral shaft fractures and vice versa, in terms of long-term functional outcome [5]. The choice between conservative and surgical treatment has traditionally been multifactorial, influenced by age and weight of the child, associated injuries, the fracture characteristics, institutional or surgeons' preferences, economic and social concerns [5-7]. Due to rapid bone remodelling, most of the femoral fractures in children younger than six years can be managed conservatively by traction and plaster-cast immobilization [8]. After six years of age, femoral shaft fractures in particular, managed non-operatively may be complicated by loss of reduction, mal-union and poor school attendance [3, 8]. Thus, the best treatment option between 6 and 15 years of age is controversial [2, 8-10]. Over the last two decades, there has been a preference for surgical reduction of these fractures in children older than six years of age [6, 8]. This preference stems from early mobilization, shorter hospital stay and avoidable detrimental psychological and financial losses experienced by patients and members of their families [2, 3, 8]. Moreover, the use of flexible intramedullary nailing has revolutionized the treatment of paediatric femoral fractures by improving on cost-effective results; early union due to repeated micro-motion at fracture site, respect for the physeal plates, early ambulation, minimal scaring, easy implant removal and tremendous patient satisfaction [8, 11]. However, this surgical option coupled with other invaluable health care infrastructures like fluoroscopy are still inexistent in many low-income countries, hindering efforts for current goal standard surgical reduction of paediatric femoral fractures [11]. Few studies have been published on paediatric femoral fractures in resource-constraint environments like the sub-Saharan African region [11, 12]. Hence, we proposed this study to determine the therapeutic patterns, treatment challenges and outcomes of the treatment of paediatric femoral fractures in the surgical unit of a tertiary center of Cameroon. The research goal is to provide some evidence which may guide clinicians make informed decisions in their choices of therapeutic strategies for paediatrics femoral fractures in resource-limited settings.

## MATERIALS AND METHODS

We carried out a prospective cohort study of all cases of paediatric femoral fractures managed between 2011 and 2015 at the surgical unit of Yaoundé University Teaching Hospital, Cameroon. Patient inclusion criteria were an age younger than 16 years; treated for non-pathological femoral fractures and followed-up for a year in the aforementioned hospital; informed consent given by the patient, parents or guardians; adequate radiological documentation. Using a structured questionnaire, all consenting consecutive participants were recruited on admission, and then examined clinically and radiologically in less than 30 minutes. Treatment was surgical or non-surgical depending on the patient’s age, weight, associated injuries, the location and pattern of the fracture, economic and social concerns. Variables studied were demographique parameters, mechanisms of injury, clinical and radiological findings confirmatory of femoral fracture, fracture classification by the AO system [13] or Salter Harris classification where appropriate, details of treatment adopted, duration of immobilization and length of hospital stay. Additionally, we studied therapeutic challenges pertaining to the availability and type of anaesthetic management, availability of an intra-operative fluoroscopy and financial constraints of parents or guardians. The outcome of treatment at 12 months of follow-up was evaluated by fracture consolidation time and the occurrence of complications such as compartment syndrome, cast contact dermatitis, bed sores, angular deformity, non-union, limb length discrepancy, infection, refracture, amyotrophy or knee ankylosis

All patients underwent periodic clinical and radiological evaluation at intervals of 2, 6, 8, 12 and 24 weeks, as necessary if there was angulation post initial fracture reduction or suspected malunion. For operative treatment, weight bearing was allowed when the construct was stable. Following non-operative management, weight bearing was allowed at cast removal. The outcome of treatment was assessed as excellent, good or bad using the Flynn’s criteria [11] Table **1**.

The data obtained were entered into Epi info 3.5.1 statistical software. All the variables were distributed in the form of simple frequencies. Means of numerical variables were reported and the threshold for statistical significance set at 0.05. Patients loss of follow-up were excluded from the final analysis.

## RESULTS

### General Characteristics of Participants and Fracture Details

During the study period, we managed 30 femoral fractures from 29 children representing 20% of all paediatric fractures managed in our hospital. These were 18 males and 11 females with a sex ratio of 1.6. Their mean age was 4.2 ± 3.3 years with a median of 3 years. Their ages ranged from 4 days to 13 years and the most represented age group was 0-5 years (76%). The mechanisms of injury were eleven (38%) accidental falls, nine (31%) road traffic accidents, seven (24%) game injuries, and two (7%) obstetrical injuries (Table **2**). Four patients presented with associated injuries to the head and clavicule; humerus; tibia; soft tissues. All fractures were closed. The affected femoral bone segments in decreasing frequencies were; 23 diaphyseal, five proximal and two distal femoral segments.

### Therapeutic Patterns

**Fig. (1):**
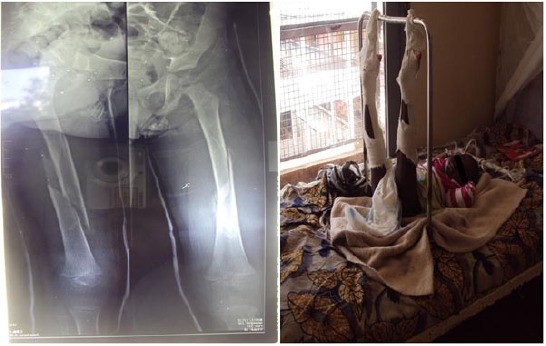
Fractures of both femurs in a three-year old child managed by skin traction followed by casting.

**Fig. (2):**
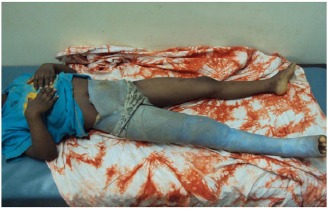
A spiral femoral shaft fracture managed by a single-leg spica cast in a five-year old child.

**Fig. (3):**
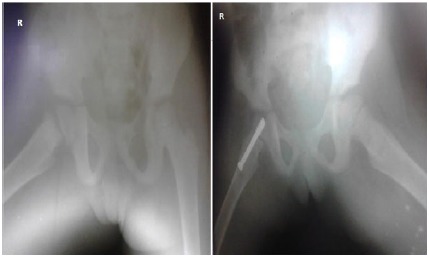
Delbet type I fracture (right) in an 8-year-old girl treated by cannulated screw fixation (left).

Twenty-one (72.4%) fractures were managed within 24 hours of injury. The fracture line in diaphyseal fractures was spiral in 14 (60.9%) cases, oblique in six (26.1%) cases, transverse in two (8.7%) cases and communited in one (4.3%) case. Twenty six (86.7%) fractures were managed by non-operative or conservative methods namely; 12 by traction followed by plaster cast immobilisation (Fig. **1**), 10 by plaster cast immobilization alone (Fig. **2**) and four by closed reduction followed by casting. Surgical fixations involved open reduction and cannulated screws fixation in two cases (Fig. **3**), pin fixation in one case and one case of external fixation.

### Therapeutic Challenges

Therapeutic difficulties encountered during orthopaedic reductions were lack of sedation anaesthesia for all the 12 traction procedures and 8 out of the 10 closed reduction procedures, and unsatisfied parents by prolonged periods of immobilization of their kids. Therapeutic difficulties encountered during surgical reductions were: lack of an image intensifier fluoroscopy to cross-check anatomical re-alignment of fractures and financial constraints of parents which compelled the conversion of three surgical indications to non-operative management.

### Outcome of Treatment

**Fig. (4):**
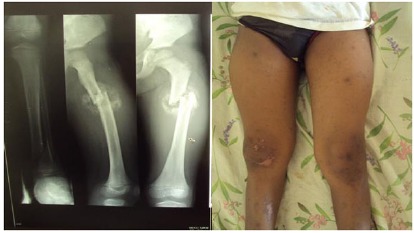
Angular deformity and mal-union following management by skeletal traction for an oblique laterally displaced proximal diaphyseal femoral fracture (with an associated distal tibial fracture) in a 13-year old child with financial constraints.

The mean duration of radiologic consolidation was 10.3 ± 3.9 weeks (range 5 - 23 weeks). Using Flynn’s criteria, the outcome of treatment was rated excellent in 28 (93.3%) cases, good in one (3.4%) and poor in one (3.4%) case. The “poor” outcome was an angular deformity of 12 cm with mal-union and residual pain at 12 months following skeletal traction a femoral shaft fracture in a 13 year old adolescent with financial constraints. The other complication was a limb length discrepancy of 1.5 cm following external fixation of a communited femoral fracture, assessed as a “good” outcome using Flynn’s criteria (Fig. **4**). We did not observe any case of compartment syndrome, non-union, rotational deformity, infection, refracture, amyotrophic or knee ankylosis. Children managed by conservative methods had longer durations of hospitalization, fracture immobilization and consolidation compared to those managed by surgery, though statistically insignificant (Table **3**). Longer immobilizations (p < 0.0001) and consolidation time (p < 0.0001) was ob-served in school age children (older than 5 years) compared to preschool children (younger than 6 years old) (Table **4**).

## DISCUSSION

Femoral fractures account for 20% of paediatrics fractures admitted in our surgical department. From the current literature, they represent less than 2% of fractures in children [1]. This may be explained by the fact that other types of paediatric fractures are less disabling in nature than femoral fractures, thus, often considered benign by the child’s parents who tend to consult more traditional healers than health care centers for fracture management in our settings. Also, under-estimation of the true incidence of paediatric femoral fractures by the current literature also seems likely. These fractures pose a significant public health problem in low-income countries like Cameroon with increasing transport activities of commercial motor bikes, largely responsible for road traffic accidents in our cohort. Paediatric femoral fractures affect more boys (62.1%) than girls (37.9%), explained by the turbulent nature of boys and resultant high-risk play activities. This finding is consistent with that from other African series [12, 14, 15]. The mean age of children with femoral fractures was 4.2 ± 3.3 years and the most affected age group was 0-5 years (76%) explained by the fact this age group does not yet possess matured cognitive and perceptuo-motor abilities to avoid accidental injuries [15, 16]. As such, their physical strength outweighs judgment, and protective reflexes are not fully developed making them a high risk group for femoral fractures [1]. This young mean age in our study may equally reflect the absence of parental awareness or education and the tendency of children to play at home or in in-secured playing grounds unsupervised. Other authors reported higher mean ages varying between due 6.8 - 7.5 years [3, 14].

The literature describes the mechanisms of injury of femoral fractures in children as age-dependent [1, 3, 4]. The main mechanisms in our series were unintentional injuries from falls (38%) and games (24%), consistent with our mean age of 4.2 ± 3.3 years and findings obtained in South Africa by Mughal *et al.* [15]. This is in contrast to other studies [12, 14, 17] which reported road traffic accident as the major aetiology in 56.7 - 68.8% of cases, explained by their relative more active and older study population with a mean ages varying between 6.5 to 12.1 years.

There is no consistency regarding fracture line presentations from the literature [1, 3, 4]. We found 60.9% spiral, 26.1% oblique, 8.7% transverse and 4.3% communited fractures. Buechsenschuetz *et al.* found that 35.2% of the fractures were oblique, 35.2% transverse, 16.9% spiral and 12.6% comminuted [18].

Non-operative management was the mainstay of treatment in 26/30 cases and operative treatment in 4/30 cases. The indications of operative or non-operative management used in our series were similar to those described by several authors [6, 7]. In our study, the low mean age of participants (4.2 ± 3.3 years), the low rate of associated injuries (4/29 children) and the high proportion of closed simple diaphyseal non displaced fractures (23/30 cases) were already highly suggestive that there should be greater indication of a conservative approach in fracture management. Although with low quality evidence, a recent meta-analysis of randomized controlled trials concluded that compared to conservative treatment, elastic intramedullary nailing may shorten rehabilitation time [5]. The benefits of a speed up recovery cannot be over emphasized, given our observed longer durations of immobilization (p < 0.0001) and consolidation (p < 0.0001) in school-aged children compared to preschool children. Using Flynn’s criteria, the results of treatment were excellent in 25/26 cases (96.2%) managed by non-operative methods and excellent in 3/4 cases (75%) managed operatively, with comparable durations of hospitalization, fracture immobilization and consolidation. This finding is of major economic significance in our resource-constrained setting where many parents cannot afford surgical management for their injured children. As reported by other African authors [11, 14, 17], we encountered infrastructural challenges from our health care setting as well as parental financial constraints which precluded optimal management of our patients. Measures which reduce hospital stay like home traction for toddlers and a brief period of traction followed by casting may curb these therapeutic challenges. Also, means for elastic intramedullary nailing and image intensifier fluoroscopy, should be put at the disposal of centers managing these fractures. While implementation of a national health insurance may ensure that injured children are being given the appropriate timely treatment and help resolve parental financial constraints.

We acknowledge some drawbacks of our study; its small sample size (n=30) and single study setting. As such, our findings may be generalized to the entire nation with caution. However, based on well followed-up patients, we have used a cohort design to provide a contribution of level II scientific evidence to the scarcity of data on the treatment, challenges and outcomes of paediatric femoral fractures in the sub-Saharan African region. These findings may guide clinicians making informed decisions in their therapeutic strategies for paediatrics femoral fractures in resource-challenged environments.

## CONCLUSION

Our findings suggest that one out of every five paediatrics fractures encountered at our surgical department, are femoral fractures. Affected children are often younger than six years and boys are more affected than girls. Parents need to be sensitized on preventable aetiologies so as to be more vigilant. The treatment of these fractures by conservative methods yields favourable outcomes. This is of great economic interest in our resource-limited setting. However, modern evidence-based surgical techniques like elastic intramedullary nailing are needed for early rehabilitation, a better patient-parent satisfaction and prevent poor school attendance of injured school-aged children. Good quality multi-center randomized controlled trials comparing conservative versus surgical interventions for treating paediatric femoral fractures in sub-Saharan Africa are needed.

## Figures and Tables

**Table 1:** Flynn’s criteria for assessment of treatment.

**Features**	**Excellent**	**Good**	**Poor**
Limb length discrepancy	< 1 cm	< 2 cm	>2 cm
Angulation in degree	< 5 cm	5 - 10 cm	>10 cm
Pain	Absent	Absent	Present
Complication	Absent	Minor and resolved	Major complication and/or lasting morbidity

**Table 2:** Characteristics of paediatric femoral fractures managed.

**Case**	**Age**	**Sex**	**Mechanism of injury**	**AO** **class**	**Other injuries**	**Mode of treatment**	**Type of anaesthesia**	**Complication**	**Outcome**
1	5 years	M	Game	3.2.A.1.2	None	conservative	None	None	Excellent
2	2 years	M	Fall	3.2.A.1.2	None	conservative	None	None	Excellent
3	3 years	M	Fall	3.3.A.1.1	None	conservative	None	None	Excellent
4	22 months	M	Game	3.2.A.2.2	None	conservative	None	None	Excellent
5	3 years	M	Fall	3.2.A.1.3	None	conservative	None	None	Excellent
6	5 years	M	RTA	3.2.A.1.2	Humerus	conservative	GA	None	Excellent
7	3 years	F	Fall	3.2.A.1.2	None	conservative	GA	None	Excellent
8	4 years	M	Game	3.2.A.1.1	None	conservative	None	None	Excellent
9	3 years	M	Game	3.2.A.1.1	None	conservative	None	None	Excellent
10	11 months	F	RTA	3.2.A.1.2	None	conservative	None	None	Excellent
11	4 days	M	Birth injury	3.2.A.1.2	None	conservative	None	None	Excellent
12	2 years	F	Fall	3.2.A.2.1	None	conservative	None	None	Excellent
13	12 years	M	RTA	3.2.A.2.1	None	conservative	None	None	Excellent
14	8 years	F	Fall	3.1.A.1.2	None	conservative	None	None	Excellent
15	8 years	M	RTA	3.1.A.2.1	Head + Clavicle	conservative	None	None	Excellent
16	3 months	F	Fall	3.2.A.2.2	None	conservative	None	None	Excellent
17	15 months	M	RTA	3.3.A.2.2	None	conservative	None	None	Excellent
18	6 years	F	Game	3.2.A.1.2	Tibia	conservative	None	Angular Deformity + Malunion	Poor
19	2 years	M	Fall	3.2.A.1.2	None	conservative	None	None	Excellent
20	2 years	M	Game	3.2.A.2.2	None	conservative	None	None	Excellent
21	3 years	M	Fall	3.2.A.1.2	None	conservative	None	None	Excellent
21bis	3 years	M	Fall	3.2.A.1.2	None	conservative	None	None	Excellent
22	13 days	M	Birth injury	3.2.A.2.2	None	conservative	None	None	Excellent
23	3 years	M	RTA	3.2.A.1.2	None	conservative	None	None	Excellent
24	8 years	F	RTA	3.2.C.1.2	Soft Tissues	OREF	GA	Limb Length Discrepancy	Good
25	13 years	F	RTA	3.1.A.3.2	None	ORIF^b^	GA	None	Excellent
26	5 years	F	Game	3.1.A.1.1	None	ORIF^b^	GA	None	Excellent
27	5 years	F	Fall	3.2.A.3.1	None	conservative	None	None	Excellent
28	3 years	M	Fall	3.2.A.2.2	None	conservative	None	None	Excellent
29	8 years	F	RTA	Salter Harris I	None	ORIF^a^	GA	None	Excellent

RTA = Road traffic accident; GA=general anaesthesia; OREF= Opend reduction and external fixation; ORIF^a^= Opened reduction and internal fixation with cannulated screws; ORIF^b^ = Opened reduction and internal fixation with pins.

**Table 3:** Comparison of conservative and surgical treatment.

**Variables**	**Non operative** **treatment N=26**	**Operative** **Treatment N=4**	**p value**
Means of Length of hospital stay (weeks)	3.0±1.1	2.7±1.2	0.6191
Means of immobilization (weeks)	7.8±3.4	7.3±2.3	0.7799
Means of consolidation (weeks)	10.4±4.2	9.3±2.3	0.6161

**Table 4:** Comparison of conservative treatment by age groups amongst the 23 cases.

Variables	0-5 years N=23	6-15 years N=7	p value
Means of Length of hospital stay (weeks)	3.0±.1.2	3.0±.0.7	1.000
Means of immobilization (weeks)	6.9±2.8	12.3±1.1	**< 0.0001**
Means of consolidation (weeks)	9.3±3.0	15.8±2.5	**< 0.0001**

## LIST OF ABBREVIATIONS

AO: = Arbeitsgemeineschaft fur Osteosynthesefragen
PFF: = Paediatric femoral fracture
